# Progression of Mortality due to Diseases of the Circulatory System and Human Development Index in Rio de Janeiro Municipalities

**DOI:** 10.5935/abc.20160141

**Published:** 2016-10

**Authors:** Gabriel Porto Soares, Carlos Henrique Klein, Nelson Albuquerque de Souza e Silva, Glaucia Maria Moraes de Oliveira

**Affiliations:** 1 Instituto do Coração Edson Saad, Rio de Janeiro, RJ - Brazil; 2 Programa de Pós-Graduação em Cardiologia - Universidade Federal do Rio de Janeiro, Rio de Janeiro, RJ - Brazil; 3 Universidade Severino Sombra, Vassouras, RJ - Brazil; 4 Escola Nacional de Saúde Pública - Fiocruz, Rio de Janeiro, RJ - Brazil

**Keywords:** Cardiovascular Diseases/mortality, Brain Diseases/mortality, Epidemiology, Economic Indexes, Social Conditions, Social Indicators, Censuses

## Abstract

**Background:**

Diseases of the circulatory system (DCS) are the major cause of death in Brazil and worldwide.

**Objective:**

To correlate the compensated and adjusted mortality rates due to DCS in the Rio de Janeiro State municipalities between 1979 and 2010 with the Human Development Index (HDI) from 1970 onwards.

**Methods:**

Population and death data were obtained in DATASUS/MS database. Mortality rates due to ischemic heart diseases (IHD), cerebrovascular diseases (CBVD) and DCS adjusted by using the direct method and compensated for ill-defined causes. The HDI data were obtained at the Brazilian Institute of Applied Research in Economics. The mortality rates and HDI values were correlated by estimating Pearson linear coefficients. The correlation coefficients between the mortality rates of census years 1991, 2000 and 2010 and HDI data of census years 1970, 1980 and 1991 were calculated with discrepancy of two demographic censuses. The linear regression coefficients were estimated with disease as the dependent variable and HDI as the independent variable.

**Results:**

In recent decades, there was a reduction in mortality due to DCS in all Rio de Janeiro State municipalities, mainly because of the decline in mortality due to CBVD, which was preceded by an elevation in HDI. There was a strong correlation between the socioeconomic indicator and mortality rates.

**Conclusion:**

The HDI progression showed a strong correlation with the decline in mortality due to DCS, signaling to the relevance of improvements in life conditions.

## Introduction

The Human Development Index (HDI), a measure of long and healthy life, access to education and standard of living, comprises three main pillars, health, education and income. The HDI was developed in 1990 by two economists, the Pakistani Mahbub ul Haq and the Indian Amartya Sen, aimed at being a general and concise measure of human development.^1,2^ Initially, health was measured as life expectancy at birth, education, as adult literacy and schooling rate, and income, as per capita Gross Domestic Product (pcGDP). That indicator was calculated as the geometric mean of its three components. From 2009 on, there have been changes: the 'education' component began being measured as the mean years of schooling and expected years of schooling, and the 'economic' component began being measured as per capita income.^3^

In Brazil, the HDI data of the municipalities were first published in 1998 retrospectively based on data of the 1970, 1980 and 1991 demographic censuses.^4^ In 2003 and 2013, new reports were published with data from the 2000 and 2010 censuses, respectively. That indicator comprises the Atlas of Brazilian Human Development,^5^ organized and made available by three institutions, the United Nations Development Programme (UNDP),^1^ the Brazilian Institute of Applied Research in Economics (IPEA),^6^ and the João Pinheiro Foundation.^7^

According to data from 1979 on, the mortality rates due to diseases of the circulatory system (DCS) and their major subgroups, cerebrovascular diseases (CBVD) and ischemic heart diseases (IHD), showed a progressive reduction in Rio de Janeiro State municipalities.^8^ The health conditions of the populations are determined in a complex way by social components, such as income distribution, wealth and level of knowledge.^9^

This study aimed at correlating the mortality rates due to DCS, CBVD and IHD with the evolution of HDI in Rio de Janeiro State municipalities.

## Methods

Data on HDI and mortality in Rio de Janeiro State municipalities were collected. The Rio de Janeiro State municipalities were constituted according to the geopolitical structure of 1950, grouping together emancipated municipalities from that date on with their original headquarters. Those aggregations of municipalities implicated in a reduction in the total number of municipalities existing in 2010 in the Rio de Janeiro State, passing from 92 to 56 aggregates for the purpose of this study analysis.

In addition, those aggregations of municipalities were grouped into regions proposed by the Rio de Janeiro State Health Secretariat with the dismemberment of the metropolitan region into the Metropolitan Belt, comprising all municipalities in the region, except for the municipalities of Rio de Janeiro and Niterói, which began constituting two other autonomous regions. The other regions, Middle-Paraíba, Mountain, Northern, Seaside, Northwestern, Southern-Central and Baía da Ilha Grande are those same defined by the Rio de Janeiro State Health Secretariat.^10^

The HDI data were obtained from the IPEA site^6^ for the years of the 1970, 1980 and 1991 demographic censuses. To estimate the HDI of the headquarter municipalities, respecting the Rio de Janeiro State geopolitical structure in 1950, arithmetic means weighted by population size of each emancipated municipality were estimated. This can be exemplified as follows: headquarter-municipality HDI = [(population of emancipated municipality A x HDI) + (population of emancipated municipality B x HDI)] / (population of emancipated municipality A + population of emancipated municipality B). The population data were retrieved from the Brazilian Institute of Geography and Statistics^4^ for the years of general census (1991, 2000 and 2010) and by counting, and were obtained in the DATASUS-MS site.^11^

The mortality rates derived from the analysis of the death data of adults aged at least 20 years and were obtained in the DATASUS-MS site.^11^ Such data were discriminated in the fractions of major interest of the study: DCS, corresponding to the codes listed in chapter VII of the International Statistical Classification of Diseases and Related Health Problems, ninth revision (ICD-9), ^12^ or in chapter IX of ICD-10;^13^ IHD, corresponding to the codes 410-414 of ICD-9 or I20-I25 of ICD-10; CBVD, corresponding to the codes 430-438 of ICD-9 or I60-I69 of ICD-10. In addition, ill-defined causes (IDC) of death were assessed, contemplated in chapter XVI of ICD-9 and chapter XVIII of ICD-10, as was the total number of deaths due to all causes. The ICD-9 was in effect up to 1995, and ICD-10 has been in effect since 1996. Raw and adjusted, for sex and age, mortality rates per 100,000 inhabitants were calculated by using the direct method.^14^ Because the mortality rates due to IDC in Rio de Janeiro State have increased significantly since 1990,^15^ we chose to use compensation, which consisted in adding to the number of declared deaths due to a specific cause a certain number of deaths due to IDC, which corresponds to the proportion of specific deaths in relation to the total of deaths. For example, if 30% of the specified deaths are due to DCS, 30% of deaths due to IDC are added to those due to DCS. Compensation was performed for all years in the series. For the deaths due to DCS, IHD and CBVD, part of the deaths due to IDC were added, corresponding to the fractions observed in the defined deaths, that is, excluding those due to IDC. After compensating deaths due to DCS, IHD and CBVD for IDC, mortality rates adjusted for sex and age were estimated. The standard population for the adjustments was that of Rio de Janeiro State counted in the 2000 census, stratified into seven age groups (20-29 years; 30-39 years; 40-49 years; 50-59 years; 60-69 years; 70-79 years; and 80 years and older) for each sex. Such rates were denominated compensated and adjusted. The mortality rates for the 1991 and 2000 census years were calculated by use of 3-year moving means and 2-year moving means for the year 2010.

Dispersion graphs were built with the mortality rates due to DCS, IHD and CBVD as ordinates, and the HDI figures as abscissae, with discrepancy of two demographic censuses. The mortality rates of the years 1991, 2000 and 2010 were related to the HDI figures of the years 1970, 1980 and 1991, respectively. In addition, the Pearson correlation^16^ of DCS, CBVD or IHD with HDI figures with discrepancy of two demographic censuses was calculated. In addition, we estimated the linear regression coefficients and R^2^ of the linear regression models with DCS, CBVD or IHD as dependent variables and HDI as independent variable, the latter with 0.1 units. We chose to consider the discrepancy of two demographic censuses based on the results of a previous study, in which mortality rates were correlated with pcGDP, and the optimal temporal discrepancy between the mortality rates and the socioeconomic indicator was, on average, close to 20 years; therefore, a similar discrepancy was adopted for this study.

Quantitative procedures were performed with Excel-Microsoft^17^ and STATA^18^ programs.

## Results

**Figure 1:**
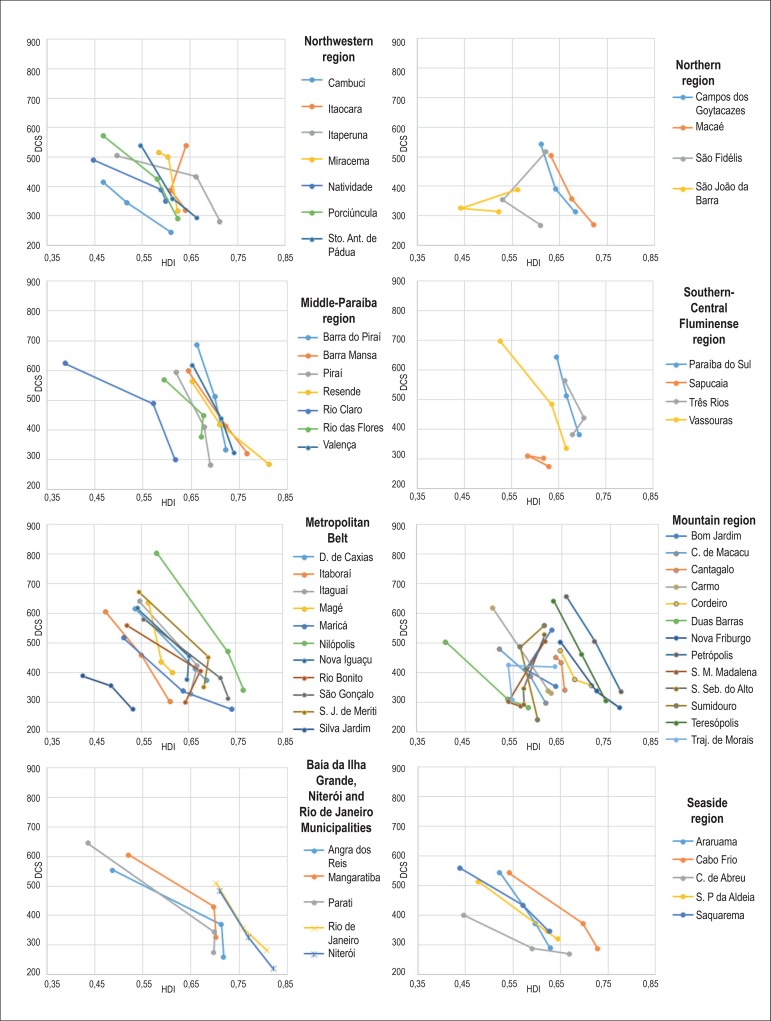
Mortality per 100,000 inhabitants due to diseases of the circulatory system (DCS) in census years of 1991, 2000 and 2010, according to the human development index (HDI) in census years of 1970, 1980 and 1991.

**Figure 2:**
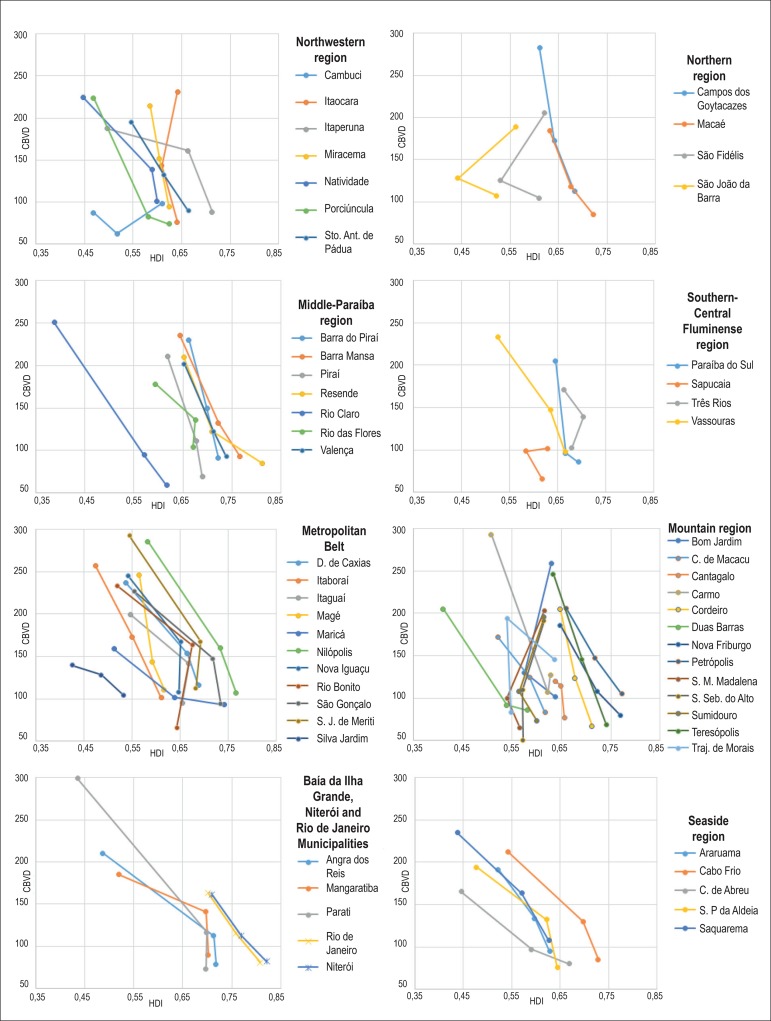
Mortality per 100,000 inhabitants due to cerebrovascular diseases (CBVD) in census years of 1991, 2000 and 2010, according to the human development index (HDI) in census years of 1970, 1980 and 1991.

**Figure 3:**
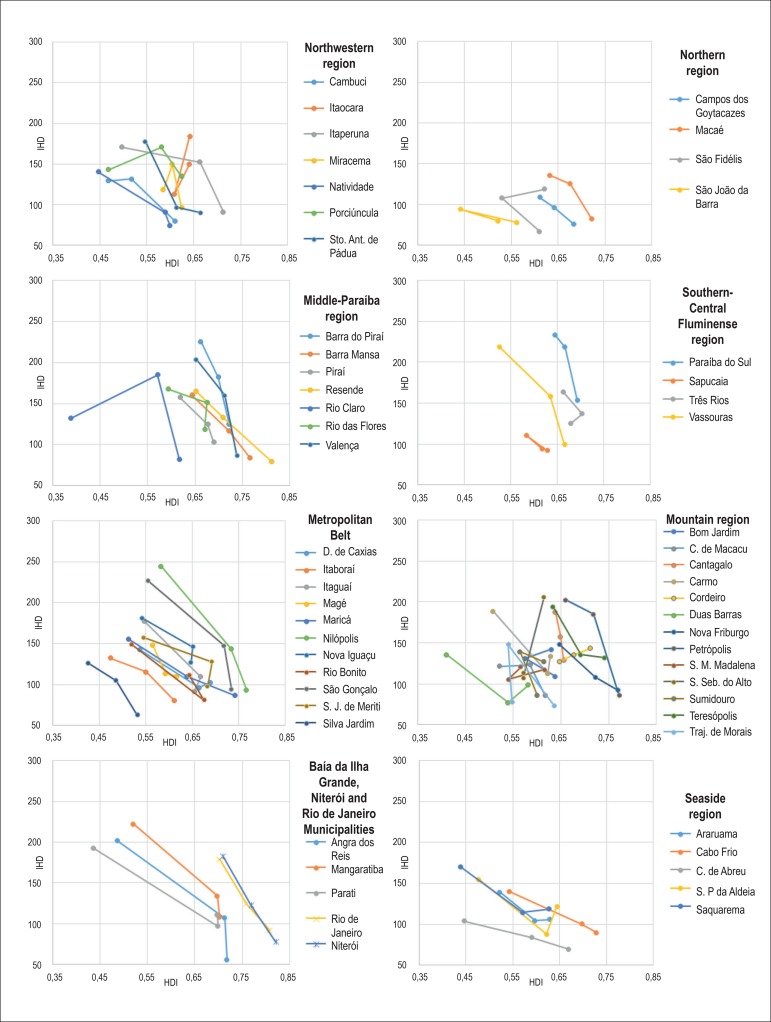
Mortality per 100,000 inhabitants due to ischemic heart diseases (IHD) in census years of 1991, 2000 and 2010, according to the human development index (HDI) in census years of 1970, 1980 and 1991.

Figures 1, 2 and 3 show an increase in HDI in most Rio de Janeiro State municipalities according to the 1970, 1980 and 1991 demographic censuses. Only seven municipalities (Itaocara, Santa Maria Madalena, São Fidélis, São João da Barra, São Sebastião do Alto, Sumidouro and Trajano de Morais) had a decrease in HDI.

All municipalities had a reduction in the mortality rates due to DCS, CBVD and IHD when comparing the initial rates (1991) with those in 2010 (Figures 1, 2 and 3).

**Figure 4:**
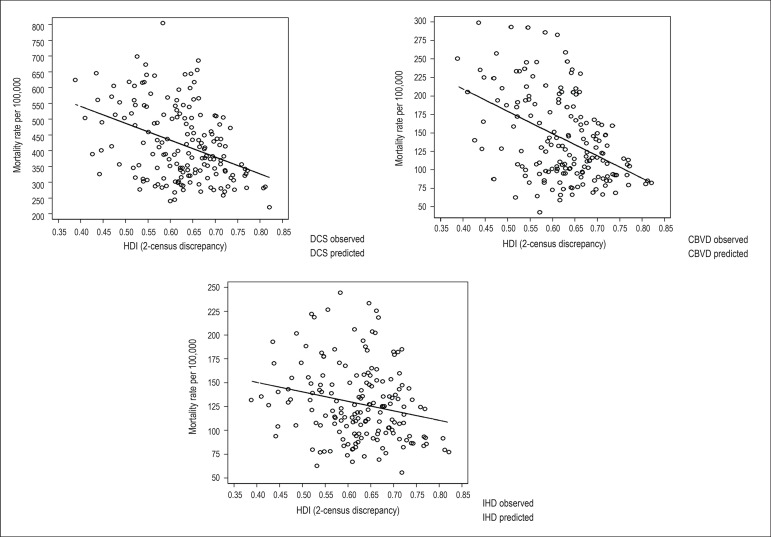
Dispersion graphs and linear adjustment of mortality rate per 100,000 inhabitants due to diseases of the circulatory system (DCS), cerebrovascular diseases (CBVD) and ischemic heart diseases (IHD) in census years of 1991, 2000 and 2010, according to the human development index (HDI) in census years of 1970, 1980 and 1991.

There is a negative correlation of mortality rates with HDI figures, the HDI increase relating to a reduction in the mortality rates due to those three causes analyzed. The correlation coefficient between the mortality rates and HDI, when calculated with the total set of municipalities (Table 1), was closer to the minimum extreme value for CBVD (-0.45), followed by DCS (-0.39) and IHD (-0.22). The R^2^ value, which explains how much of the variability of mortality can be caused by the HDI, was higher for CBVD (0.20), followed by DCS (0.15) and IHD (0.05). In addition, Table 1 shows a measure of reduction in mortality due to DCS, CBVD and IHD for each 0.1 increase in the HDI figure, described by the coefficient of linear regression. The dispersion graphs (Figure 4) show greater inclination of the line and smaller dispersion of the points in relation to the line for CBVD, while smaller inclination and greater dispersion in relation to the line occurred for IHD, DCS showing an intermediate pattern, although closer to that of CBVD.

**Table 1:** Pearson correlation coefficient, linear regression coefficient* and R2 of the relationship between mortality per 100,00 inhabitants due to diseases of the circulatory system (DCS), cerebrovascular diseases (CBVD) and ischemic heart diseases (IHD) in census years of 1991, 2000 and 2010, with human development index (HDI) with discrepancy of 2 demographic censuses, in Rio de Janeiro State municipalities

	Corr	Coef*	R^2^
DCS	-0.39	-53.5	0.15
CBVD	-0.45	-30.2	0.20
IHD	-0.22	-10.0	0.05

* 0.1 unit of HDI

Corr: Pearson correlation coefficient; Coef: linear regression coefficient.

This study showed that the reduction in the mortality rates due to DCS, CBVD and IHD in the Rio de Janeiro State in the past decades was preceded by an increase in HDI, with significant numbers, because a 0.1 increment in HDI correlated with the following reductions in the number of deaths per 100,000 inhabitants: 53.5 for DCS; 30.2 for CBVD; and 10.0 for IHD.

Although the values of the correlation coefficients were not very close to the minimum extreme limit, in the individualized analysis of the municipalities, and regarding DCS, 47 municipalities showed correlation coefficient with HDI lower than -0.7, and only 9 municipalities had it higher than -0.7: Bom Jardim, Itaocara, Santa Maria Madalena, Sumidouro, São Fidélis, São João da Barra, São Sebastião do Alto, Trajano de Morais and Três Rios. Regarding CBVD, 11 of the 56 municipalities had correlation coefficients with HDI higher than -0.7: Bom Jardim, Cambuci, Itaocara, Santa Maria Madalena, Sapucaia, Sumidouro, São Fidelis, São João da Barra, São Sebastião do Alto, Trajano de Morais and Três Rios. Regarding IHD, 10 municipalities had correlation coefficients with HDI higher than -0.7: Bom Jardim, Itaocara, Miracema, Porciúncula, Santa Maria Madalena, Sumidouro, São Fidélis, São Sebastião do Alto, Trajano de Morais and Três Rios. Of the total, only 8 municipalities (Bom Jardim, Itaocara, Santa Maria Madalena, Sumidouro, São Fidélis, São Sebastião do Alto, Trajano de Morais and Três Rios) had correlation coefficients higher than -0.7 for the three groups of causes of death (Table 2).

**Table 2:** Distribution of the Rio de Janeiro State municipalities according to Pearson correlation coefficient (inferior to or superior to -0.7) and to the diseases of the circulatory system (DCS), cerebrovascular diseases (CBVD) and ischemic heart diseases (IHD) in census years of 1991, 2000 and 2010, with human development index with discrepancy of 2 demographic censuses

DCS		CBVD		IHD	
**< -0,7**	**> -0,7**	**< -0,7**	**> -0,7**	**< -0,7**	**> -0,7**
Angra dos Reis	Bom Jardim	Angra dos Reis	Bom Jardim	Angra dos Reis	Bom Jardim
Araruama	Itaocara	Araruama	Cambuci	Araruama	Itaocara
Barra do Piraí	S. Maria Madalena	Barra do Piraí	Itaocara	Barra do Piraí	Miracema
Barra Mansa	São Fidélis	Barra Mansa	S. Maria Madalena	Barra Mansa	Porciúncula
Cabo Frio	S. João da Barra	Cabo Frio	Sapucaia	Cabo Frio	S. Maria Madalena
Cachoeiras de Macacu	S. Sebastião do Alto	Cachoeiras de Macacu	São Fidelis	Cachoeiras de Macacu	São Fidélis
Cambuci	Sumidouro	Campos dos Goytacazes	S. João da Barra	Cambuci	S. Sebastião do Alto
Campos dos Goytacazes	Trajano de Morais	Cantagalo	S. Sebastião do Alto	Campos dos Goytacazes	Sumidouro
Cantagalo	Três Rios	Carmo	Sumidouro	Cantagalo	Trajano de Morais
Carmo		Casimiro de Abreu	Trajano de Morais	Carmo	Três Rios
Casimiro de Abreu		Cordeiro	Três Rios	Casimiro de Abreu	
Cordeiro		Duas Barras		Cordeiro	
Duas Barras		Duque de Caxias		Duas Barras	
Duque de Caxias		Itaboraí		Duque de Caxias	
Itaboraí		Itaguaí		Itaboraí	
Itaguaí		Itaperuna		Itaguaí	
Itaperuna		Macaé		Itaperuna	
Macaé		Magé		Macaé	
Magé		Mangaratiba		Magé	
Mangaratiba		Maricá		Mangaratiba	
Maricá		Miracema		Maricá	
Miracema		Natividade		Natividade	
Natividade		Nilópolis		Nilópolis	
Nilópolis		Niterói		Niterói	
Niterói		Nova Friburgo		Nova Friburgo	
Nova Friburgo		Nova Iguaçu		Nova Iguaçu	
Nova Iguaçu		Paraíba do Sul		Paraíba do Sul	
Paraíba do Sul		Parati		Parati	
Parati		Petrópolis		Petrópolis	
Petrópolis		Piraí		Piraí	
Piraí		Porciúncula		Resende	
Porciúncula		Resende		Rio Bonito	
Resende		Rio Bonito		Rio Claro	
Rio Bonito		Rio Claro		Rio das Flores	
Rio Claro		Rio das Flores		Rio de Janeiro	
Rio das Flores		Rio de Janeiro		S. Antônio de Pádua	
Rio de Janeiro		S. Antônio de Pádua		São Gonçalo	
S. Antônio de Pádua		São Gonçalo		S. João da Barra	
São Gonçalo		São João de Meriti		São João de Meriti	
São João de Meriti		São Pedro da Aldeia		São Pedro da Aldeia	
São Pedro da Aldeia		Saquarema		Sapucaia	
Sapucaia		Silva Jardim		Saquarema	
Saquarema		Teresópolis		Silva Jardim	
Silva Jardim		Valença		Teresópolis	
Teresópolis		Vassouras		Valença	
Valença				Vassouras	
Vassouras					

All municipalities with correlation coefficients between mortality rates and HDI greater than -0.7 have small populations, less than 40,000 inhabitants in 2000, except for São João da Barra and Três Rios, which had less than 100,000 inhabitants. Together, their population does not add up to 10% of that of the Rio de Janeiro State.

## Discussion

During the 20th century, mainly after World War II, all developed countries, and a little later, the developing countries, showed improvement in their socioeconomic indicators, followed by a decline in mortality rates,^19,20^ mainly a reduction in the deaths due to DCS.^21^

Several studies have shown an inverse correlation of HDI with mortality due to neoplasms,^22,23^ infectious diseases^24^ and CBVD,^25^ or even HDI to be a predictor for other indicators, such as child and maternal mortalities.^26^ Thus, HDI elevations are related to a reduction in the number of deaths due to several causes. Even general mortality relates directly to HDI, because, one component of that indicator is life expectancy at birth, thus, if there is a reduction in the number of deaths due to any cause, there is HDI increase.

Socioeconomic improvements preceded the decline in mortality due to cardiovascular diseases, which account for almost half of the deaths due to endogenous causes in adults.^21^ The Rio de Janeiro State municipalities have heterogeneous HDI figures: in 1970, some municipalities, such as Duas Barras, Parati, Rio Claro and Silva Jardim, had HDI close to 0.4, comparable to that of Ethiopia and Mozambique. Other municipalities, such as Rio de Janeiro, Niterói and Resende, had much higher HDI, closer to that of Scandinavian countries.^2^ Several municipalities that began the series with low HDI figures showed HDI elevations in the following years of census, and a progressive reduction in cardiovascular mortality, showing that not only HDI figures, but its progressive improvement, relate to a reduction in mortality rates.

The main limitation of this study relates to the availability of HDI figures for municipalities only from the census years of 1970 onward. That is compounded by the fact that those figures were calculated retrospectively for all years, and there was a change in the calculation method from the year 2009 onward.^3^ This study used only the HDI values calculated for the 1970, 1980 and 1991 census years, because such figures were homogeneous regarding the calculation method. From the year 2000 onwards, other calculation methods began to be used for the estimations, with changes in the HDI components; therefore, they were not used in this study. Other limitation relates to the fact that some municipalities had small populations, less than 40,000 inhabitants, being subject to significant oscillations regarding the annual occurrence of any low-frequency event, such as death. In an attempt to minimize that occurrence, the mortality rates were calculated by using 3-year moving means for all aggregations of municipalities. Other limitations derive from the use of mortality data subject to the influence of factors such as improper completion of death certificates and the compensation maneuver for IDC, which might have underestimated or overestimated the deaths due to defined causes.

In recent decades, there was a significant reduction in the mortality rates of Rio de Janeiro State municipalities due to DCS, especially CBVD, and that reduction was preceded by periods of elevation in HDI. This shows the important correlation of HDI with the reduction in those mortality rates, signaling to the relevance of improvements in life conditions.
